# Simultaneous effects of leaf irradiance and soil moisture on growth and root system architecture of novel wheat genotypes: implications for phenotyping

**DOI:** 10.1093/jxb/erv290

**Published:** 2015-06-18

**Authors:** Kerstin A. Nagel, David Bonnett, Robert Furbank, Achim Walter, Ulrich Schurr, Michelle Watt

**Affiliations:** ^1^CSIRO Plant Industry, GPO Box 1600, Canberra, ACT, 2601, Australia; ^2^Institute of Bio- and Geosciences, IBG-2: Plant Sciences, Forschungszentrum Jülich GmbH, 52425 Jülich, Germany; ^3^ Present address: Bayer Crop Science, 90th Street S, Sabin, MN 56580, USA; ^4^ Present address: ANU College of Medicine, Biology and Environment, Australian National University, Canberra, ACT, 2601, Australia; ^5^ Present address: Institute of Agricultural Sciences, Swiss Federal Institute of Technology Zurich (ETHZ), Universitätstrasse 2, 8092 Zürich, Switzerland

**Keywords:** Light, root branching, root depth, root partitioning, water deficit, water uptake.

## Abstract

Plants in the field are exposed to varying light and moisture. Agronomic improvement requires knowledge of whole-plant phenotypes expressed in response to simultaneous variation in these essential resources. Most phenotypes, however, have been described from experiments where resources are varied singularly. To test the importance of varying shoot and root resources for phenotyping studies, sister pre-breeding lines of wheat were phenotyped in response to independent or simultaneous exposure to two light levels and soil moisture profiles. The distribution and architecture of the root systems depended strongly on the moisture of the deeper soil layer. For one genotype, roots, specifically lateral roots, were stimulated to grow into moist soil when the upper zone was well-watered and were inhibited by drier deep zones. In contrast, the other genotype showed much less plasticity and responsiveness to upper moist soil, but maintained deeper penetration of roots into the dry layer. The sum of shoot and root responses was greater when treated simultaneously to low light and low soil water, compared to each treatment alone, suggesting the value of whole plant phenotyping in response to multiple conditions for agronomic improvement. The results suggest that canopy management for increased irradiation of leaves would encourage root growth into deeper drier soil, and that genetic variation within closely related breeding lines may exist to favour surface root growth in response to irrigation or in-season rainfall.

## Highlight

This paper demonstrates the value of simultaneously varying shoot and root resources for future phenotyping studies by revealing phenotypic differences of wheat genotypes exposed to multiple light and moisture conditions.

## Introduction

Plants use multiple resources by shoots and roots simultaneously. Few phenotyping studies, however, vary the supply of more than one resource to plants, and it is unclear if phenotypic information from single-factor treatments is relevant to plants in field environments (reviewed in Rich and Watt, 2013). Recent studies have revealed that the response of plants to a combination of two different abiotic stresses (like drought and heat) is unique and cannot be directly extrapolated from the response of plants to each of the different stresses applied individually (Rizhsky *et al.*, 2002; Mittler, 2006). Chapman *et al.* (2011), for example, showed that responses in roots measured under different nitrate supply were removed when mild water stress was co-applied.

This paper explores the importance of independently or simultaneously varying shoot and root resources for phenotyping studies. Shoots and roots of sister pre-breeding lines of wheat (*Triticum aestivum*) were phenotyped in response to independent or simultaneous exposure to two light levels and soil profiles: a moist top soil with a dry or moist deeper soil. Wheat plays a major role in nutrition of the world’s population and is grown on more than 200 million hectares of land worldwide (FAO 2013; http://faostat.fao.org/site/567/default.aspx#ancor). Wheat production per year must increase about 70% by 2050 to meet population demands and avoid price rises (Fischer and Edmeades, 2010). Current gains in wheat through conventional yield-based breeding are too slow. Unlike the other major cereals, maize (*Zea mays*) and rice (*Oryza sativa*), the wheat genome has not been sequenced. Phenotyping is therefore expected to play an important role in speeding up yield gains by breeders (Hall and Richards, 2013).

Light to the shoots for photosynthesis, and soil water to roots for cell expansion and transpiration are arguably the most critical resources required by plants (Boyer, 1982). While responses of plants to light regimes or soil water conditions have been extensively investigated independently of each other, responses of plants to simultaneous variation in these resources are not well understood. According to the model of ‘functional equilibrium’, plants respond to a decrease in above-ground resources (such as light) with increased allocation to shoots (leaves), whereas they respond to a decrease in below-ground resources (such as water) with increased allocation to roots (Brouwer, 1963). Plants shift their biomass allocation so that the plant can capture more of the limited resources, resulting in maximal growth rate under given environmental conditions (Poorter and Nagel, 2000). However, there are differing predictions about whether a given soil moisture availability has a stronger impact than leaf irradiance on the vegetative growth of shoots and roots. To date, there have only been a few studies that deal with the combination of drought and light treatments, and these have shown species-specific results: low light (under high soil water content) induced the dry-matter allocation to the shoot of castor bean (*Ricinus communis*; Penfound, 1932) and *Picea* seedlings (Yang *et al.*, 2008) but reduced shoot biomass of *Amomum villosum* (Feng and Li, 2007) and wheat (Tamaki *et al.*, 2001). Likewise, diverse reactions have been found under low light in combination with low soil moisture in terms of reduced biomass partitioning to roots. It is well known that some species may grow their roots to penetrate deeper if the soil dries, but the mechanism for this is unclear (Sharp and Davies, 1985). Yet, it is also conceivable that the flexible generation of shallow roots can confer advantages in drought stress: root systems would better utilize the potential of seasonal, short rainfall events that often saturate only a thin layer of top soil (Prechsl *et al.*, 2015). Boyer *et al.* (2010) showed that phloem water could supply root growth into dry soil, suggesting that higher light intensities would promote the growth of roots into dry soil, and that less root penetration into dry soil would be expected under lower light conditions. As far as could be determined, there have been no phenotyping studies of how light and soil moisture simultaneously alter development of leaf area, root extension, or root system architecture.

Here, phenotyping of roots and shoots from two novel wheat genotypes, sister lines developed in a pre-breeding programme to incorporate greater shoot vigour into wheat (Richards and Lukacs, 2002), was performed. Shoot and root growth and architecture monitoring were combined with measurements of water-use efficiency (WUE). A soil profile was designed with a well-watered top soil and low soil water content in the bottom part of a rhizobox to study the effect of spatially distributed water availability on root architecture, while independently or simultaneously applying two light levels to leaves. The difference in phenotypes due to independent or simultaneous variation of leaf light and soil water could then be measured, as well as the extent to which pre-breeding sister lines exhibited different phenotypes in response to these shoot and root conditions.

## Materials and methods

### Plant material

*Triticum aestivum* pre-breeding lines VJ 10 and VJ 30 were used. These wheat lines are progeny of a cross between Vigour 18 (V), developed from a high specific leaf area parent and a large embryo parent (Richards and Lukacs, 2002), and cultivar Janz (J). Janz was a successful variety that was widely adapted to climates and soils in Australia in the 1990s and 2000s, but has lower leaf and root vigour than V 18 (Watt *et al.*, 2005; Liao *et al.*, 2006). The pedigree of the VJ lines used was Vig18/2*Janz8-28 (Janz8-28 = HM14BS/2*Janz). They were produced by single seed descent after the final cross, and selected for vigour based on leaf width. They are taller and earlier maturing than Janz. The height is probably because they have the reduced height (*Rh*) gene *Rht8*, and lack *Rht1* from Janz (*Rht8* is weaker than *Rht1* but does not reduce coleoptile length or vigour, Ellis *et al.*, 2004). VJ 10 and VJ 30 reach grain development faster than Janz, perhaps owing to the presence of *ppd-D1* linked to *Rht8*. VJ 10 and 30 are very similar to one another in height, flowering time, and general shoot appearance so they are a good pair for vigour comparisons both at early stages and later plant growth stages. Before starting the study, preliminary analysis was conducted on the leaf and root vigour of VJ 10 and 30. They were found to have differences in partitioning between roots and shoots when grown in tall columns of sandy soil to five leaves (methods in Watt *et al.*, 2008).

### Rhizoboxes and soil moisture treatments

**Fig. 1.**
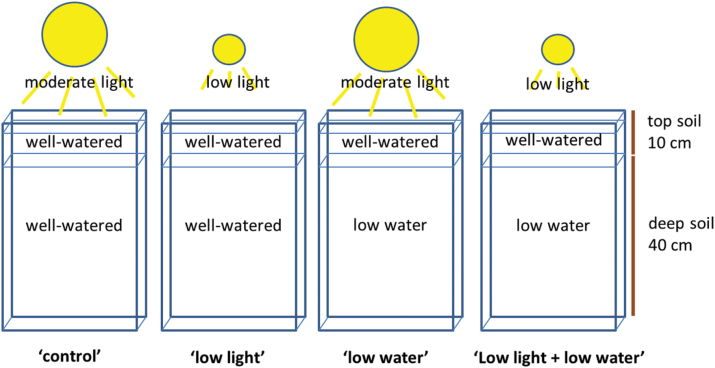
Schematic of four growth conditions applied to two wheat genotypes. Light regimes: moderate light (450 µmol m^−2^ s^−1^ photosynthetically active radiation) and low light (250 µmol m^−2^ s^−1^ photosynthetically active radiation). Water conditions: well-watered (0.14g g^−1^) and low water (0.06g g^-1^) (this figure is available in colour at *JXB* online.)

VJ 10 and VJ 30 plants were cultivated in rhizoboxes (10×250×500mm) filled with a mixture of 50% sand and 50% potting soil, sieved 3mm (see Fig. 1 for rhizobox set-up used here; adapted from Refshauge *et al.*, 2006; Boyer *et al.*, 2010). Seeds were pushed 3cm deep into the soil embryo facing downwards—two seeds per rhizobox—positioned at the transparent surface of the rhizobox, which was then covered with black foil. The black cover was only removed for root growth measurements. The top soil (top 0–10cm) was filled with soil with a water content of 0.14g g^−1^. The bottom soil (from top 10cm to 50cm) was set either to 0.14g g^−1^ (well-watered) or to 0.06g g^−1^ (low water). The soil at the top of the rhizobox was covered with a transparent film with holes for the shoots to prevent evaporation. Water content of the soil was maintained three times per week by weighing the rhizoboxes and adding lost water with a syringe from the top through the small holes in the transparent film. The supply of water to rhizoboxes from the top is a standard procedure in phenotyping experiments because this is the practical way to keep soil moisture at a controlled level. Addition of water from below is difficult to monitor and apply precisely. Application of water to the top soil could lead to situations in which the top layer of the rhizobox has higher moisture than deeper layers, but this pattern of water application to soil is common in the field during short irrigation events (Benli *et al.*, 2007) or rain showers (Prechsl *et al.*, 2015).

In a pre-experiment, a protocol for the application of water from the top was established to maintain the added water in the top layers of the rhizobox. The water was added in small portions of maximum 5mL with a syringe through the holes for the shoots in the transparent film. The water movement was checked visually by colour changes of the soil at the transparent face of the rhizoboxes. After stabilization of the water movement the next portion of water was added until the target weight of each rhizobox was reached again. By using this protocol the water content in the top layer of the rhizoboxes could be maintained over the whole experiment (maximum shift of the border between both layers was ±1cm). This was confirmed by taking soil samples at harvest.

The plants were illuminated either in steady-state light conditions at 450 (moderate light) or 250 (low light) µmol m^−2^ s^−1^ photosynthetic active radiation. After sowing, the rhizoboxes were set in an angle of approximately 45°, with the clear face facing downwards. Plants were treated at day/night temperatures of 18°C/20°C (±1°C) and 12h/12h light/dark cycles in a growth cabinet (Conviron, Canada).

In summary, plants were treated with four different combinations of soil moisture conditions and light regimes (shown in Fig. 1):

‘control’ – moderate light combined with well-watered conditions;

‘low light’ – low light combined with well-watered conditions;

‘low water’ – moderate light combined with low water conditions;

‘low light + low water’ – low light combined with low water conditions.

The experiment was conducted in a complete randomized design of the two genotypes, four conditions, with two plants per rhizobox and four biological replicates per genotype and treatment. The position of the rhizoboxes in the growth chamber was randomly changed at each measurement time point to reduce the effect of local differences in environmental conditions (such as temperature and air humidity).

### Leaf and root growth measurements

**Fig. 2.**
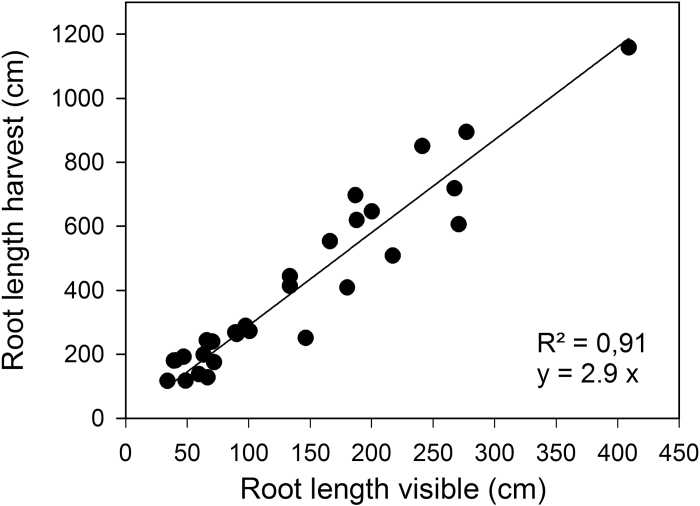
Ratio between visible and non-visible root system of wheat plants grown in soil-filled rhizoboxes. Root length visible at the transparent surface of the rhizoboxes is plotted against the total root length after harvesting the plants.

Three times per week, the number of main stem leaves and tiller leaves was counted, and the length and width of all leaves measured with a ruler. The measurement was started 10 days after sowing, when the first leaf was unrolled. In total, the leaves were measured at five time points. The total leaf area (A) was then calculated according to A = leaf width × leaf length × 0.858 (Kalra and Dhiman, 1977). The measurement of roots started 5 days after sowing, when the first roots were visible at the transparent surface of the rhizoboxes. In total, the roots were measured at seven time points. Three times per week, the number of lateral roots arising from the primary seminal roots was counted, and the root depth measured as the vertical distance between the seed and the deepest root tip. The total root length (sum of seminal and lateral root length) was measured non-invasively by tracing the roots visible at the transparent surface of the rhizobox. The roots were first traced on transparency film, and the root length was then determined by scanning the film and analysing with WinRhizo software (WinRhizo, Regent Instrument). The visible root length at the surface of the rhizobox represented part of the total root system length. To establish the effect of different light and soil moisture treatments on all of the root length, it was necessary to define the relationship between visible and non-visible roots. To do this, plants were harvested, roots washed out, and root length determined with WinRhizo. The visible root length represented approximately 30% of the total root system length, which is consistent with published data about the root fraction growing on the glass face of rhizoboxes (Hurd, 1963; Nagel *et al.*, 2012). The root length visible at the rhizobox surface and the total root length (visible and non-visible roots) showed a strong correlation (R^2^ = 0.91, Fig. 2). Hence, the non-destructive analysis of the root length at the rhizobox surface is a reliable measure of the total root length, and the effects of shoot and root treatments.

Measurements of shoots and roots were stopped at 20 days after sowing because the roots of plants grown under control conditions had reached the bottom of the rhizoboxes. This was found for both genotypes, which had similar germination times and similar progression through developmental stages to the time of harvest.

### Statistical analysis

The effect of leaf irradiance and soil moisture on leaf and root growth was analysed using a two-way ANOVA (SigmaStat, Systat Software Inc., Richmond, CA, USA). A combined ANOVA was carried out to test the effect of treatments and genotypes, and the interaction effects using a linear mixed model. *Post hoc* comparisons of treatment effects were performed within each group using the Tukey adjustment. Two-way ANOVA for repeated measures over time was used to analyse the time by treatment interactions (JMP, Version 8, SAS Institute Inc., Cary, NC, USA).

## Results

Results of the entire study are summarized in Table 1. Here, first addressed are the results for VJ 10 shoot and root growth upon varying leaf light and soil moisture through the profile independently and simultaneously, and then comparisons among responses to all treatments between genotype VJ 10 and VJ 30 are given.

**Table 1.** Summary table of the effect of irradiation and soil moisture on shoot and root growth as well as WUE of wheat line VJ 10 (black arrows) versus wheat line VJ 30 (white arrows) plants 20 days after germination.

	Low light	Low water	Low light **+** low water	*P*-value genotype	*P*-value treatment	*P*-value genotype and treatment
Leaf area	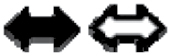	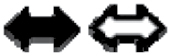	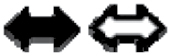	0.169	0.074	0.701
Root length top soil	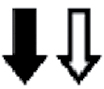	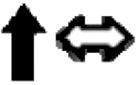	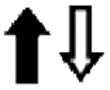	0.002	<0.001	<0.001
Root length bottom soil	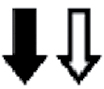	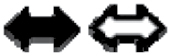	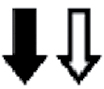	0.505	<0.001	0.112
Root depth	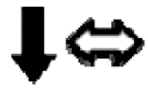	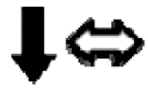	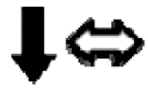	0.023	<0.001	0.009
WUE	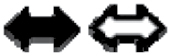	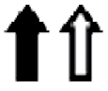	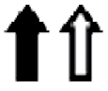	0.638	<0.001	0.994

Downward arrows represent a significant reduction, upward arrows a significant induction, and horizontal arrows no effect in growth parameters (leaf area, root length in the top and bottom part of the rhizoboxes, and root depth) as well as in WUE under a reduction of light (low light) or soil moisture in the bottom part of the rhizoboxes (low water), or a combination of low light and soil moisture (low light + low water) compared to plants grown under control conditions (moderate light and well-watered), respectively. A two-way ANOVA was used as a statistical test and *P*-values for the two factors, genotype and treatment, as well as the interaction between them, are presented in the table.

### Do light and water supply influence leaf and root system development?

**Fig. 3.**
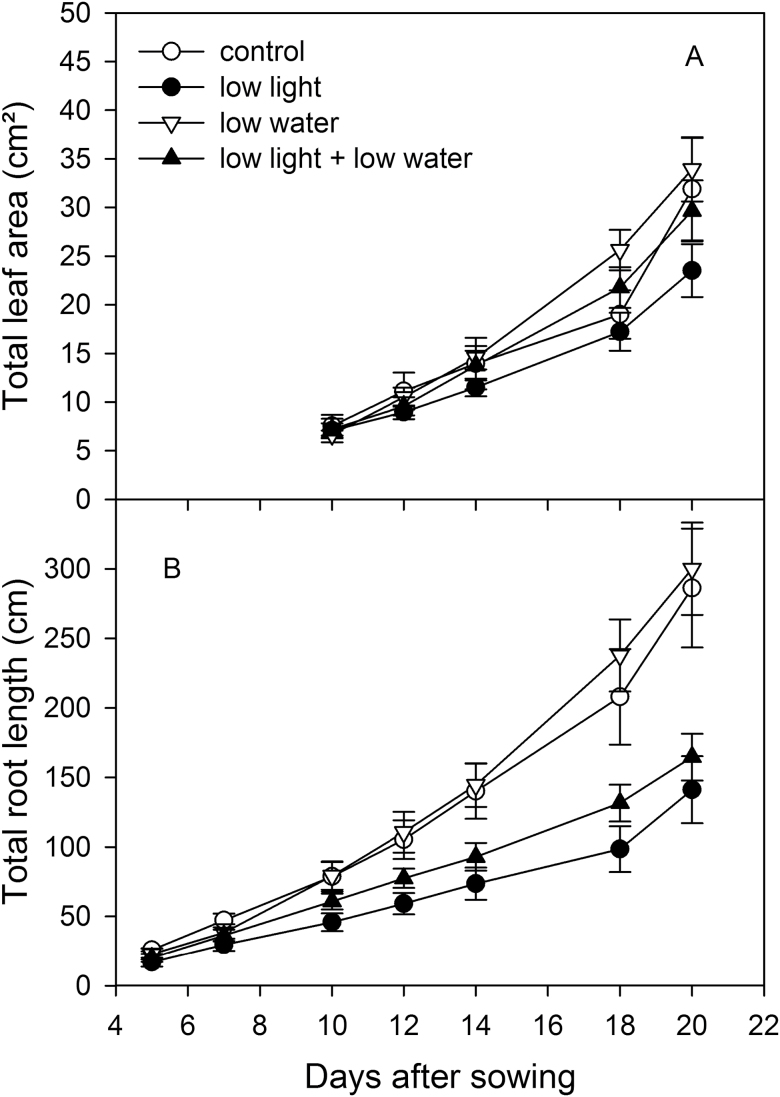
Effect of irradiation and soil moisture on (A) leaf area development and (B) total length of visible roots of wheat genotype VJ 10. Plants were exposed to control conditions (moderate light and well-watered) or to a reduction in either light (low light) or soil moisture in the bottom part of the rhizoboxes (low water), or to a combination of low light and soil moisture (low light + low water), respectively (mean value ±SE, n = 4; two-way ANOVA for repeated measures over time, (A) *F*_12,48_ = 2.07, *P* = 0.04; (B) *F*_18,90_ = 8.21, *P* < 0.001).

Leaf area development of the novel genotype VJ 10 was slightly but not significantly reduced when light irradiation was halved (Fig. 3A). The small light-dependent leaf area reduction was more pronounced when roots were exposed to well-watered conditions than to low soil water content. A decrease in light intensity led to a decrease in leaf area size of 26% under well-watered conditions, but only 13% under low soil moisture. Reduced light intensity was also found to reduce leaf numbers on main stem and tillers by approximately one leaf per plant (Table 2). Interestingly, the length of the second stem leaf increased by 20–30% when the light irradiation was halved, while width of the mentioned leaf declined slightly. Low soil water conditions induced marginally greater leaf growth, but this was not statistically significant, and numbers of leaves on the main stem were similar under low and high soil moisture (Table 2).

**Table 2.** Effect of irradiation and soil moisture on number of stem and tiller leaves, leaf length, and leaf width of the second leaf of VJ 10 wheat plants measured 20 days after sowing.

	Control	Low light	Low water	Low light **+** low water
Leaf number	5.0±0.0	3.7±0.2	5.0±0.0	4.0±0.0
Tiller number	1.0±0.3	0.0±0.0	2.4±0.6	0.0±0.0
Second leaf length (cm)	16.6±0.6	20.3±1.0	17.2±0.4	22.4±1.1
Second leaf width (mm)	3.67±0.09	3.38±0.15	3.58±0.17	3.42±0.08

Plants were exposed to control conditions (moderate light and well-watered) or to a reduction in either light (low light) or soil moisture in the bottom part of the rhizoboxes (low water), or to a combination of low light and soil moisture (low light + low water), respectively (mean value ±SE, n = 4).

The root extension rate of VJ 10 plants was slower with low light intensity applied to leaves (Fig. 3B). This amplified over time, up to a reduction of 51% under well-watered conditions and about 45% under low soil moisture conditions at day 20 after germination (Fig. 3B, *P* < 0.001). Low soil moisture stimulated root growth slightly, but not significantly compared to plants grown under well-watered conditions.

Therefore, shortest root systems were found in plants grown under low light with well-watered conditions, and longest root systems in plants grown under higher light intensity. Leaf irradiation effects were relatively independent of soil moisture conditions. Hence, because low light intensity did not change leaf growth significantly but reduced root growth strongly, light treatment led to a change in plant partitioning and root to shoot ratio (Fig. 3).

**Fig. 4.**
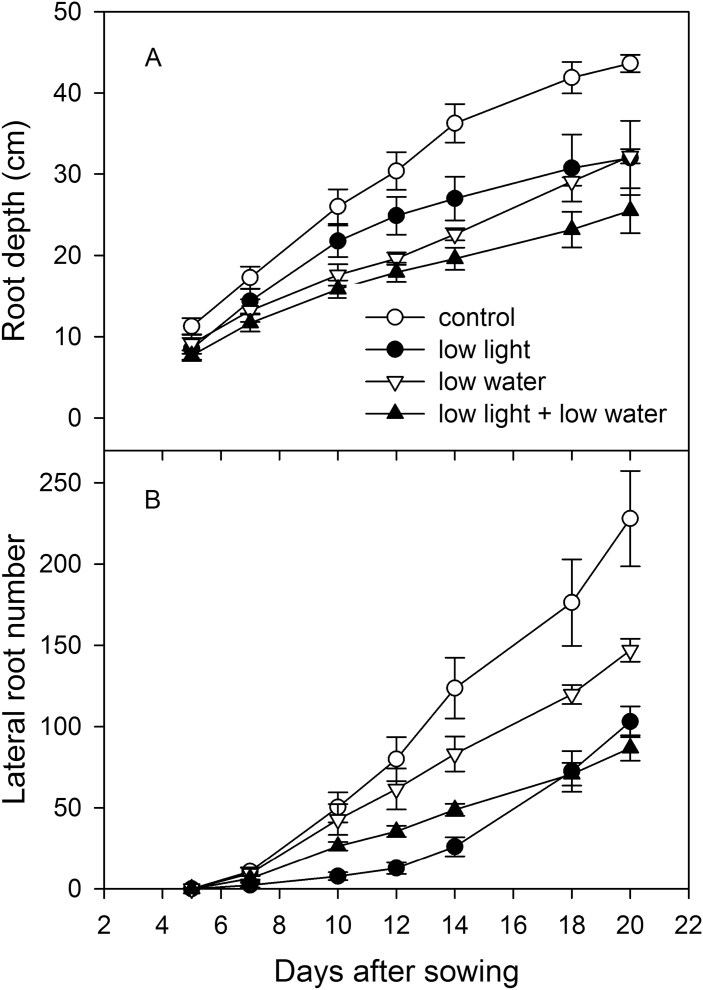
Effect of irradiation and soil moisture on (A) root depth and (B) number of lateral roots of VJ 10. Plants were exposed to control conditions (moderate light and well-watered) or to a reduction in either light (low light) or soil moisture in the bottom part of the rhizoboxes (low water), or to a combination of low light and soil moisture (low light + low water), respectively (mean value ±SE, n = 4; two-way ANOVA for repeated measures over time, (A) *F*_18,90_ = 7.61, *P* < 0.001; (B) *F*_18,90_ = 2.41, *P* = 0.001).

Now examining shifts in root architecture, low light was found to diminish the maximum depth of root systems up to 27% (Fig. 4A), and diminish the number of lateral roots and total root length (Figs 3B and 4B). Reduced depth was achieved through reduced downwards penetration rate (Fig. 4A). Similarly, low soil moisture in the bottom part of the rhizobox strongly inhibited root depth by comparison to well-watered plants (Fig. 4A; *P* < 0.001). Low soil water also diminished branching of roots by up to 36% compared to well-watered conditions at day 20 (Fig. 4B; *P* = 0.001). The highest number of lateral roots was produced in plants grown under moderate light and well-watered conditions, while the lowest number of lateral roots occurred when light and soil water content were limited.

### Do light and pattern of water supply affect root growth partitioning?

**Fig. 5.**
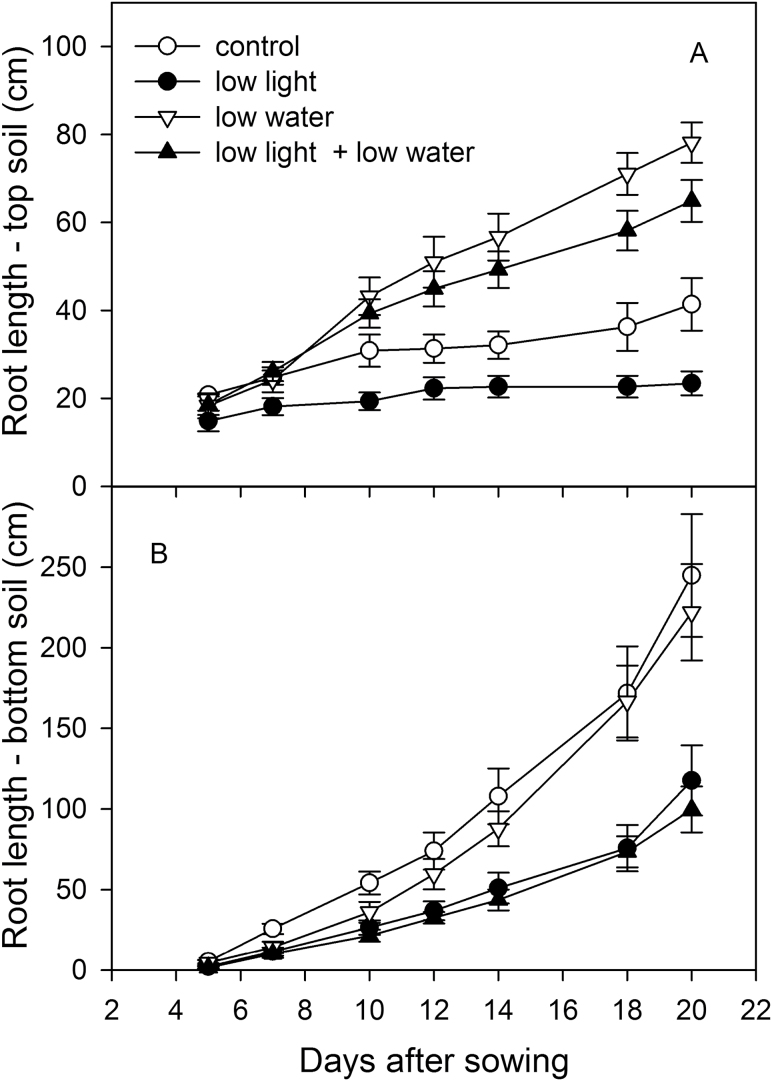
Effect of irradiation and soil moisture on root growth partitioning: root length of wheat genotype VJ 10 plants in the (A) top and (B) bottom part of the rhizoboxes. Plants were exposed to control conditions (moderate light and well-watered) or to a reduction in either light (low light) or soil moisture in the bottom part of the rhizoboxes (low water), or to a combination of low light and soil moisture (low light + low water), respectively (mean value ±SE, n = 4; two-way ANOVA for repeated measures over time, (A) *F*_18,84_ = 12.04, *P* = 0.01; (B) *F*_18,84_ = 8.66, *P* < 0.001).

As shown in Fig. 1, rhizoboxes were filled with well-watered soil in the top 10cm, but had either low or high soil water content in the bottom part. The soil moisture profile changed root partitioning between the top and bottom parts of the rhizoboxes. When the leaves were exposed to low light, the root length of the main axes and lateral roots were shorter in the top soil, which was kept under well-watered conditions (Fig. 5A). This low-light-induced reduction, however, was more pronounced (43%) if the lower compartment soil was well-watered. If the lower compartment was dry, low light around the leaves reduced root length in the top soil less, by only 17%.

Approximately 5 days after sowing, root tips reached the border between top and bottom soil compartments of the rhizobox; a further 5 days later, the first changes in root growth due to different soil water profiles were detected (Fig. 5). These changes intensified thereafter until the end of observations (*P* < 0.001). Low soil water content in the bottom compartment stimulated roots to grow in the top soil that was kept well-watered, while root growth in the lower soil with low moisture was slightly restricted (Fig. 5B): root length in the top soil increased by up to 2.8 times, but decreased by a factor of approximately 1.2 in the bottom part under low light. This induction of root growth in the well-watered soil (top soil) and inhibition in the low moisture soil (bottom soil) was more pronounced under lower light intensity around the leaves.

### Does simultaneous treatment with light and water supply alter the effects on root growth?

The question remains, when plants were treated simultaneously with both low light and low soil water at the same time, would treatment responses be additive and lead to a stronger response than to a single treatment?

**Fig. 6.**
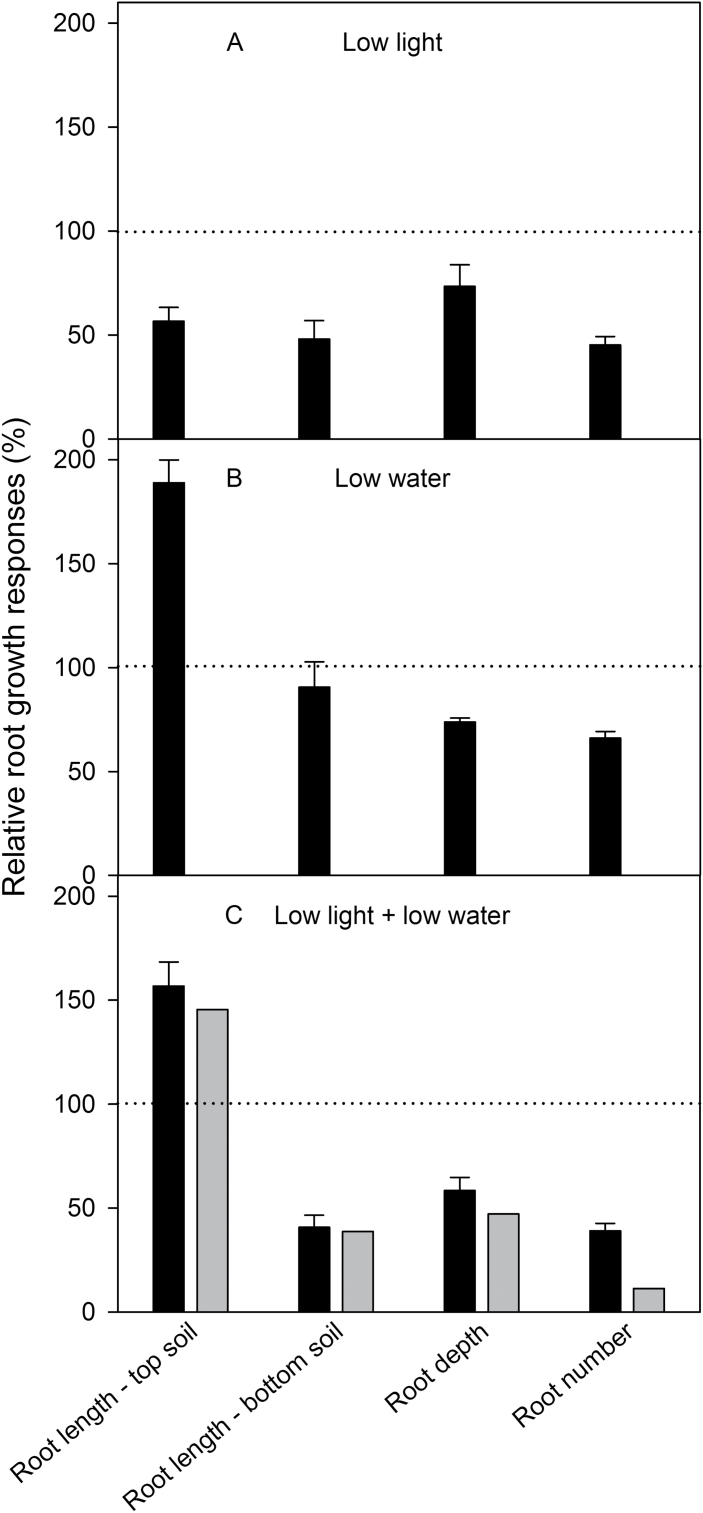
Summary of root growth responses of VJ 10 wheat plants to leaf irradiation and soil moisture 20 days after germination. Ratio between plants exposed to control conditions (moderate light and well watered) and the other three treatment combinations: (A) reduction in light intensity; (B) reduction in soil moisture; and (C) reduction in both light and soil moisture; quantified for root length in the top and bottom part of the rhizoboxes, root depth, and number of lateral roots. Values prior to plants exposed to control conditions were set to 100% (mean value ±SE, n = 4). Additionally, the theoretical root growth reduction under low light combined with low soil moisture (grey bars, C) was calculated as the sum of relative growth reductions under low light and the relative growth reductions under low soil.

Low light treatment reduced root growth in top and bottom parts of the rhizobox, but low water treatment increased root length, notably of lateral roots, in the top, well-watered soil and reduced it only slightly in the bottom soil (Fig. 6A,B). Simultaneous treatment of low light and low water in the bottom compartment also increased root length in the top part of the boxes, but to a lesser extent than in response to low water alone (Fig. 6C). In other words, extra root growth in upper layers-when the deeper layer was dry-was lower at low leaf light than higher leaf light. Simultaneous low leaf light and low soil water in the lower compartment decreased root growth more than under low light treatment alone. Expressed another way, low leaf light inhibited root growth in the lower compartment more when the soil was dry than wet.

In summary, VJ 10 plants had less root growth for all measured parameters (root length, depth, and branching) under low light, under low water, and after simultaneous treatment of low light and low moisture. The exception was greater root growth in well-watered top soil when the bottom soil had low moisture (Fig. 6). Compared to roots of plants grown under higher light and water conditions, root growth was decreased least under low soil water content (relative growth reduction: 23%); however, it was decreased at low light by twice as much (45%); and decreased further under the simultaneous treatment of both (54%). If the independent reductions by low light (45%) and low soil moisture (23%) were summed, the total growth reduction would be 68% compared to plants grown under higher light / well-watered conditions. This implies that the roots grew 14% more under low light and low soil water than would be expected from the theoretic additive value of responses to each of light and moisture independently. In other words, the whole phenotype is not a sum of the part phenotypes, and phenotypes from single treatment experiments do not necessarily predict phenotypes of multiple conditions found in environments.

### Do light and water supply affect WUE?

Responses in root and leaf development with leaf irradiation and soil water content were examined to determine if they were accompanied by changes in WUE, or water uptake rates of roots. Because water content of the rhizoboxes was controlled with periodic weighing and lost water was added from the top, it was possible to examine WUE and water uptake by roots.

**Fig. 7.**
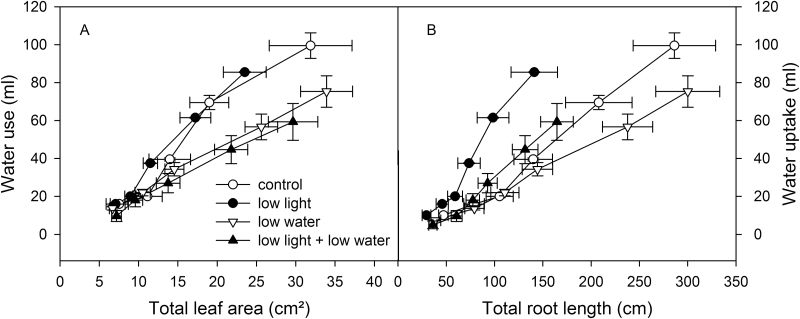
Effect of irradiation and soil moisture on WUE. (A) Total leaf area or (B) total root length are plotted against the water use of VJ 10 wheat plants. Plants were exposed to control conditions (moderate light and well-watered) or to a reduction in either light (low light) or soil moisture in the bottom part of the rhizoboxes (low water), or to a combination of low light and soil moisture (low light + low water), respectively (mean value ±SE, n = 4).

Light did not alter the WUE of VJ 10 plants. In contrast, low soil moisture in the bottom part of the rhizoboxes compared to well-watered conditions increased WUE (Fig. 7A). Consequently, plants treated with limited water supply used less water to build up a comparable leaf area size than plants grown under high soil water content. Plants grown under low soil moisture not only exhibited a 40% improved WUE, but also reduced water uptake rate per root length by approximately 40% (Fig. 7B). Under low light intensities, however, the water uptake per root length was up to 2.2 times higher. Consequently, the lowest water uptake rate per root length was found in plants exposed to higher light regimes combined with low soil water, and highest uptake values were detected in plants grown at low light combined with high soil water conditions. This led to an enhancement of water uptake by a factor of approximately 2.6 (Fig. 7B).

### Do genotypes differing in root partitioning exhibit altered reactions to light and water supply?

**Fig. 8.**
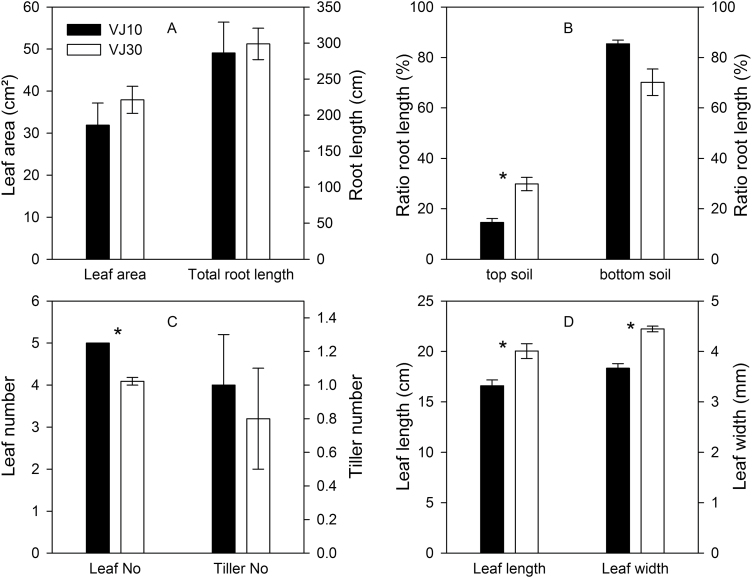
Root and leaf development (A, C, D) and root growth partitioning in different soil layers (B) of VJ 10 and VJ 30 wheat plants 20 days after germination under control conditions (moderate light and well-watered). Ratio between the root length in top or bottom soil layer and the total visible root length, respectively (mean value ±SE, n = 4). *Significant differences between the genotypes (*P* < 0.05).

The novel wheat genotype VJ 30 produced almost the same total root length under control conditions as described in the analyses above for genotype VJ 10 (Fig. 8A), but genotypes differed in their partitioning of total root length (Fig. 8B). VJ 10 produced a relatively higher amount of roots in deeper soil layers, while VJ 30 produced a relatively higher amount of roots in top soil layers (Fig. 8B).

While VJ 10 plants produced 12% higher stem and tiller leaf numbers (Fig. 8C), VJ 30 plants exhibited 21% longer and wider leaves under control conditions (Fig. 8D). Consequently, the outcome was almost the same leaf area (Fig. 8A), and light and soil moisture seemed to affect leaf growth and development similarly: a reduction in light intensity led to a slightly smaller leaf area in both genotypes, while suboptimal water supply and the combination of low light intensity with low soil moisture did not modify leaf growth (Table 1).

**Fig. 9.**
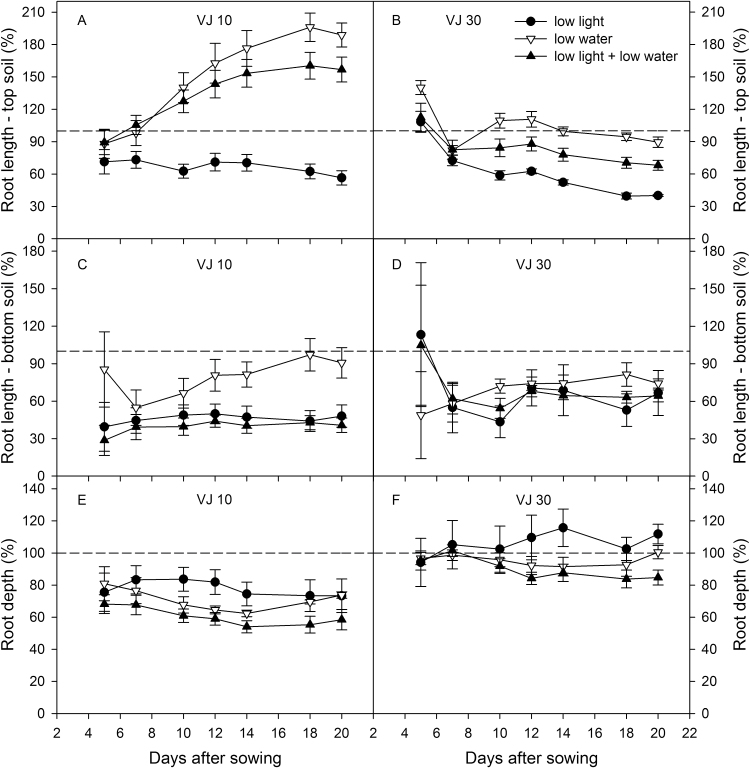
Effect of irradiation and soil moisture on root growth partitioning on VJ 10 and VJ 30 wheat genotypes. Ratio between plants exposed to moderate light and moderate soil moisture conditions and the other three treatment combinations for root length in (A, C) the top and (B, D) bottom part of the rhizoboxes. Values prior to plants exposed to control conditions (moderate light and well-watered) were set to 100% (mean value ±SE, n = 4, two-way ANOVA for repeated measures over time, (A) *F*_12,72_ = 9.14, *P* < 0.001; (B) *F*_12,108_ = 1.03, *P* > 0.05; (C) *F*_12,72_ = 2.16, *P* < 0.05; (D) *F*_12,108_ = 0.12, *P* > 0.05; (E) *F*_12,72_ = 1.75, *P* > 0.05; (F) *F*_12,108_ = 1.33, *P* > 0.05).

In spite of differences in root partitioning in control conditions, both wheat genotypes showed a similar response under low light conditions: an inhibition of root extension rates in upper as well as deeper substrate layers (Fig. 9). However, VJ 30 plants exhibited a stronger growth reduction due to light changes in the top part of the rhizobox than VJ 10 plants (VJ 30: 33% versus VJ 10: 36%). In contrast to that, low light inhibited the root growth of VJ 10 plants in the bottom part of the rhizobox 25% more than it did in VJ 30 plants in the same substrate layer.

Analysis of the effect of low soil moisture in the bottom part of the rhizoboxes on root growth showed the strongest contrast between the genotypes. Whereas limited water in the bottom significantly promoted root growth of VJ 10 in the well-watered top layers of the rhizoboxes (Fig. 9A; Table 1; *P* < 0.001), root growth of VJ 30 plants exhibited no significant differences compared to control plants (Fig. 9B; Table 1; *P* > 0.05). In contrast, although root extension in the dry bottom soil was less for both genotypes, inhibition was weaker in VJ 10 than in VJ 30 plants (VJ 10: 9% versus VJ 30: 26%; Fig. 9C,D). Under combined low light with low soil moisture conditions, VJ 10 plants had a significant root growth induction into the top substrate layers and inhibition in the bottom soil (Fig. 9A,C; Table 1). VJ 30 however showed a reduction of root growth in the deeper substrate layers and in the top soil layers (Fig. 9B,D; Table 1). In other words, VJ 30 did not show plasticity in upper soil in response to lower soil moisture. Consequently, the rooting depth of VJ 10 plants was reduced under all examined conditions, whereas VJ 30 plants exhibited no significant changes in rooting depth under low light and low soil moisture conditions, or under the treatment of both environmental factors (Fig. 9E,F, Table 1). Interestingly, despite the differences in root partitioning and the different responses to light and soil moisture conditions, VJ 10 and VJ 30 plants had similar WUE: a reduction in light intensity alone did not influence WUE, but low soil moisture and the combination of low light and low water moisture enhanced the WUE of both wheat genotypes (Table 1).

In summary, significant differences between genotypes and significant interactions among treatments and genotypes were found for root architecture, that is, root growth within the top soil and root system depth (Table 1).

## Discussion

Light exposure to the shoot and soil water availability to the roots impacted root growth of wheat plants. The combination of treatments also had an impact on root architecture and the distribution of lateral roots within the soil water profile. In the following paragraphs, the effect of leaf irradiance and soil water content on leaf and root growth will be discussed separately, and then the influence of the combined treatments is interpreted.

### Effect of leaf irradiations on leaf and root growth

Growth effects of leaf irradiation were consistent with the published data on different species under differing light regimes. These studies also report that root length and biomass increase linearly with accumulated, intercepted, photosynthetic active radiation (e.g. Vincent and Gregory, 1989; Bingham and Stevenson, 1993; Nagel *et al.*, 2006). A reduction in light intensity leads to a change in plant development and root to shoot ratio (Fig. 3, Walter and Nagel, 2006; Walter *et al.*, 2007). The strong decline of root growth under low light conditions may be triggered by a restricted translocation of leaf assimilates into the below-ground part of plants (Campbell and Read, 1968; Farrar and Jones, 2000; Freixes *et al.*, 2002). Carbohydrate deficit in this study appeared to have reduced total root system growth, including the depth, but to a lesser extent than it reduced root extension (Figs 3 and 4). Growth into deep soil layers over root extension into upper soil layers may be beneficial for wheat plants under drought stress conditions, where deeper soil layers have a higher probability of remaining moist. Root extension rate into dry soil likely depends on water carried in photoassimilates of the phloem (Boyer *et al.*, 2010); water carried in the phloem may explain how low light can reduce root elongation, in addition to limiting carbohydrate to root tips.

### Effect of spatial distribution of soil water content on leaf and root growth as well as root architecture

The reduction of below-ground water led to a slightly larger root system, which enables the plants to increase uptake of limited water and improve WUE (Fig. 7). An induction of root length and biomass by dry soil conditions as well as an increased root to shoot ratio has been reported previously (Campbell and Read, 1968; Müller-Thurgau, 1975; Passioura, 1981; Dubrovsky *et al.*, 1998; Carvalho *et al.*, 2014). The earliest events reported in drying soil were increases of root diameter, followed by a reduction in leaf elongation (Schmidhalter *et al.*, 1998). No significant changes to leaf initiation and leaf area were observed in the present study.

It is a common practice to apply drought stress to plants by drying the soil, and to apply water to plants by homogeneously watering the soil. Under natural conditions, soil can have layers with higher soil moisture than others. Therefore, a spatial distribution of soil water content was analysed, with watered top soil layers in combination with dry bottom soil layers, which can occur in the field with short irrigation or rainfall events. Non-uniform spatial distribution of soil moisture may explain the discrepancy in shoot growth behaviour in this study compared to others. Plants appear to sense the drying of the soil around roots and communicate this information to the shoot (Sauter *et al.*, 2001; Chaves *et al.*, 2003). In dry soil, roots could be sensing multiple conditions: falling availability of phosphorus, falling water status, or the hardening of the soil (Passioura and Gardner, 1990). Root signals, like abscisic acid (ABA) or other chemical, physical, or hydraulic signals (Ober and Sharp, 2003; Chaves *et al.*, 2003), can influence stomatal behaviour (and therefore carbon gain; Davies and Zhang, 1991), but also regulate partitioning of carbohydrates to roots (Karmoker and Van Steveninck, 1979). In a heterogeneous distribution of soil water content, signals from different parts of the root system presumably would be integrated to change the partitioning of roots. Here, low soil moisture in deeper soil layers strongly stimulated roots - 5 days after the first root tips had reached the dryer soil - to grow into the well-watered top soil layers (Fig. 5). The signal of roots growing in low-moisture layers may have not only led to this strong growth induction into soil layers with high water availability, but also to an increase in WUE of the shoots (Fig. 7). As a consequence of this acclimatization, the fraction of the root system that had access to water seemed to be sufficient to supply the wheat plants with adequate water and to maintain leaf extension at a high growth level (Fig. 3; Table 2).

Only VJ 10 expressed increased root length in the well-watered top soil; the VJ 30 genotype did not (Fig. 9; Table 1). To determine whether VJ 30 plants are less efficient under dry soil conditions than VJ 10 plants, whole plant responses should be compared under low soil moisture. Although VJ 10 plants produced more leaves, the leaf area of both lines was similar and, therefore, the two lines theoretically had a comparable photosynthetic potential. In spite of the similarity in shoot size, the lines differed in their strategies to cope with the heterogeneous soil water distribution. VJ 10 used the energy of imported carbohydrates to optimize the root growth in soil layers with high water availability and exhibited an increased lateral root proliferation to presumably improve water and nutrient access (Figs 4, 5 and 9). In contrast, VJ 30 plants invested fewer roots in the well-watered top soil, but sustained root growth into deeper soil layers to a similar level as under well-watered conditions (Figs 8 and 9; Table 1). In the long run, a deep penetration of roots by VJ 30 may be more efficient under drought field conditions, which will have a drying of surface soil but have water stored in deeper soil layers. Equally, it is conceivable that the development of more shallow roots in VJ 10 plants confers advantages in conditions that receive short rainfall events. It was recently shown in temperate grassland that generating a higher fraction of shallow roots in the top 10cm of the soil in drought conditions is a successful strategy (Prechsl *et al.*, 2015). Plants with such a strategy may possess a higher fitness compared to control plants, because they can use the water from short seasonal rain events that will not percolate deeply into the soil and evaporate rapidly. Therefore, the root architecture of VJ 10 might lead to an advantage for plants specifically selected for such field situations.

### Simultaneous effects of leaf irradiation and soil water content on leaf and root growth

A 50% reduction of light intensity led to a 50% shorter root system, and a 50% reduction of soil moisture content stimulated up to 2-times greater root growth in the top well-watered soil layers (Figs 3–6). Would the effects of both stress parameters be additive and lead to a stronger growth reaction than one resource limitation alone? Theoretically, if both low leaf irradiation and low soil water content are applied simultaneously, the reduction of root growth under low light, and the induction of root growth under low soil moisture could be compensated for in the top soil layers. However, the simultaneous treatment of low light and low water on VJ 10 plants increased root length in the top part, but not as strongly as the low water treatment alone (Figs 6 and 9). This result implies that there must be a significant interaction of leaf irradiation and water supply on root growth in the top well-watered layers. Both factors also affected roots in the bottom of the rhizoboxes: low light intensity as well as low soil moisture reduced root growth in the bottom, but not as strongly as under both environmental parameters (Fig. 6). In summary, a simultaneous treatment of low light and low soil moisture inhibited root extension and branching in the bottom of the rhizoboxes, which led to a 42% shorter root system (Figs 4–6). A similar reduction in root length of 43% was shown for castor bean plants (Penfound, 1932). For wheat plants, Tamaki *et al.* (2001) reported an inhibition of 68% in root biomass under combined low light and low water availability. The difference in reduction on wheat plants could be ascribed to the discrepancy in time points when plants were measured or harvested. Tamaki *et al.* (2001) harvested the plants at anthesis whereas the plants in this study were grown for only 3 weeks in rhizoboxes (because roots reached the bottom of the rhizoboxes 3 weeks after sowing and measurements were stopped). During these 3 weeks, inhibition of root extension increased from 23% to 42% and it may have increased more over plant development up to 68% at anthesis. However, Tamaki *et al.* (2001) did not quantify root growth continuously, so it is not possible to be certain.

Non-destructive quantification of root system architecture over time is required to reveal important features of root systems. The result presented here show that simultaneous treatment of low light and water supply led not only to a reduction in root length, but also to an inhibition of rooting depth and rate of lateral root initiation. This inhibition was enhanced over time, from 21% to 46% for the rooting depth, and from 32% to 61% for the branching rate (Fig. 4). Consequently, initiation of new lateral roots was more reduced than rooting depth and root growth rate under both low light and low soil moisture. The result was a modified root system architecture with relatively fewer lateral roots but longer roots. This adaptation of the geometry may improve efficiency of the root system, because roots may reach regions of soil with greater water availability, improving plant fitness and chances of survival.

Simultaneous treatment of lower light and lower water did not inhibit root growth as strongly as would be predicted from the theoretical sum of growth reactions to factors separately (Fig. 6). Light and water treatments may have led to a cross-talk between response pathways, and this could be mediated by transcription factors, protein kinase cascades, hormones, or other signals at cellular and whole plant levels (Mittler, 2006; citations therein). Limited water supply can for example trigger production of ABA in roots, which is transported to the leaves and causes stomatal closure (Chaves *et al.*, 2009). The amount of ABA reaching the stomata is regulated by the pH of the xylem sap, which is strongly affected by drought and light levels to leaves (Jia and Davies, 2007). It is also possible that drought and leaf irradiation may interact via sugars. Water deficit at the root level and light intensity at the shoot level alter the carbohydrate metabolism in plants (e.g. Pinheiro *et al.*, 2001; Nagel *et al.*, 2006). Sugars may regulate, for example, the water balance in plants by influencing the sensitivity of stomata to ABA (Wilkinson and Davies, 2002), or by functioning as osmolytes to protect the plant from drying out (Kawakami and Yoshida, 2005; Valliyodan and Nguyen, 2006). Sugars can mediate metabolic pathways activated by light and water availabilities by acting as signalling molecules, modifying gene expression and proteomic patterns (Smeekens, 1998), and therefore regulating photosynthesis and plant growth. More specifically, as discussed above, phloem carries water molecules to the root tip, and this phloem-derived water can support 50% of wheat root growth (Boyer *et al.*, 2010). The studies here support the notion that leaf photosynthesis and phloem supply to roots may influence root elongation into drying soil. In summary, the fact that the genotypes expressed differences in root growth into the dry soil suggests a genetic basis to sum responses to leaf light and root growth under drought, and that this could be selected through phenotyping.

### Implications for phenotyping

Phenotyping of plants in response to combinations of canopy and root conditions is scarce, yet under field conditions plants are naturally exposed to multiple environmental parameters. In this study, one shoot resource, leaf irradiation, and one root resource, soil moisture, were selected and the responses of two exemplarily selected pre-breeding wheat lines to independent and simultaneous variations of both environmental factors were compared. The two wheat lines responded differently to the combined light and drought conditions than would be expected from singular treatments of each. This suggests that there would be value from future phenotyping efforts that have the capability to vary and monitor multiple environmental factors. Because root phenotyping in the field is challenging, non-invasive methods, such as rhizoboxes combined with high throughput approaches, facilitate the screening of large number of genotypes under multiple combinations of environmental parameters. The simulation of combinations relevant in the field may enable a better selection of candidate genotypes under controlled environments and a better understanding of plant mechanisms under multiple conditions. This knowledge will speed up breeding programmes for crop improvement in the field.
